# Identification and Quantification of Flavanol Glycosides in *Vitis vinifera* Grape Seeds and Skins during Ripening

**DOI:** 10.3390/molecules23112745

**Published:** 2018-10-24

**Authors:** Marie Zerbib, Guillaume Cazals, Christine Enjalbal, Cédric Saucier

**Affiliations:** 1 Science Pour l’œnologie Univ Montpellier, INRA, Montpellier Supagro, 34000 Montpellier, France; marie.zerbib@umontpellier.fr; 2 IBMM, Univ Montpellier, CNRS, ENSCM, 34000 Montpellier, France; guillaume.cazals@umontpellier.fr (G.C.); christine.enjalbal@umontpellier.fr (C.E.)

**Keywords:** grape ripening, flavan-3-ol, glycoside, diglycoside, MRM, mass spectrometry

## Abstract

Monomeric and dimeric flavanol glycosides were analyzed in *Vitis vinifera* grapes and seeds during ripening. An analytical method using ultra-high performance liquid chromatography coupled with a triple quadrupole mass spectrometry (UHPLC-ESI-QQQ-MS/MS) in multiple reaction monitoring (MRM) mode was employed. Three grape varieties (Merlot, Syrah and Tannat) were chosen and grape berries were sampled at different stages of development. Ten monoglycosylated and six diglycosylated flavanol monomers were detected. Twelve monoglycosylated and three diglycosylated flavanol dimers were also detected for all three grape varieties. All diglycosides were detected for the first time in *Vitis vinifera* grapes, though some of these compounds were only detected in skins or seeds. Furthermore, the evolution of all these compounds was studied, and a decrease in monomeric (epi) catechin monoglycosides was observed during ripening for Tannat, Merlot and Syrah grape skins. The dimers would appear to accumulate in skin tissues up to mid-summer (after veraison) and decrease when grape berries reached maturity.

## 1. Introduction

Flavan-3-ols belong to the flavonoid class of phenolic compounds, otherwise known as flavanols. They may be present in plants in the form of monomers, proanthocyanidins (PA) polymers or condensed tannins. The monomeric units are usually linked in polymers through C4–C6 or C4–C8 linkages (B type dimer) and sometimes with additional C2-*O*-C5 or C2-*O*-C7 bonds (A type dimer). They are present in a wide variety of natural products [1] including chocolate and cocoa (up to 920–1220 mg/100 g), apples (up to 60 mg/100 g), grapes (up to 100 mg/100 g) and tea (up to 300 mg/infusion) [2,3]. Flavanol composition depends greatly on the biological tissue, method of cultivation, and grape variety. In *Vitis vinifera* berries, four principal units of PA are usually found: (+)-catechin, (−)-epicatechin, (−)-epigallocatechin and (−)-epicatechin gallate. Grape seeds contain greater amounts of (−)-epicatechin gallate [4] and generally of flavanols [5]. PA polymers are longer in skins than in seeds. Their average degree of polymerization (DP) ranges from three to 16 in seeds [6], whereas it varies from 10 to 40 in skins [7,8], though even higher values were measured (up to 100) [9,10].

These secondary metabolites represent a bioactive group with health properties, having a potential role in lowering the risk of cardiovascular disease and as antimicrobial and antiviral agents [2,11,12]. They are also involved in plant defense against biotic and abiotic stress [13]. In humans, improved assimilation is obtained by conjugation of the molecules with methyl, glucoronate, glucosyl or sulfate functions [4,14,15]. An in vivo study on rats also showed that intestinal absorption of catechin may be increased for catechin glucosides [16]. Catechin glycosylated conjugates may thus be of interest as components of natural food supplements.

The roles of glycosylated flavanols in plant metabolism are not yet fully understood; it has been suggested that they could be involved in PA biosynthesis [17]. In fact, the polymerization mechanism of flavanol constitutes the black box of the PA pathway, and consequently is an important topic in current Enology research [10]. Dixon and collaborators -demonstrated that the over-expression of a glucosyltransferase, UGT72L1, in the seed coat of *Medicago truncatula* induced increased accumulation of PA-like compounds, and at the same time, catalyzed the formation of epicatechin 3′-*O*-glucoside [18]. Flavanol glycosides allow the PA units to transfer into the vacuole and may constitute a PA precursor during polymerization.

Monomeric flavanol glycosides have been reported in diverse plants such as elm [19], barley [20], lentils [21], cowpea [22,23] or buckwheat [24]. Fourteen glycoside isomers of (+)-catechin, (−)-epicatechin, (−)-epigallocatechin and (−)-epicatechin gallate were detected in Merlot grape seeds and wine [25]. Dimeric forms of glycosylated flavanols are also present in plants, but some have been described in the literature; for example, 3-*O*-β-catechin-(4-α-8)-catechin-d-glucoside was identified in blackjack oak [26]. More recently, we detected, using ion-trap UPLC-MS, dimeric and monomeric (epi) catechin glycosides in grapeberries at different development stages in Merlot, Syrah and Cabernet-Sauvignon varieties [27]. In addition, a procyanidin dimer-7-*O*-diglucoside was identified in cowpea [23].

In this paper we describe the development of a new quantitative analytical strategy involving ultra-high performance liquid chromatography coupled with triple quadrupole mass spectrometry (UHPLC-ESI-QqQ-MS/MS) using the multiple reaction monitoring (MRM) method to quantify flavanol glycosides during grape ripening. This technique has already been used for the characterization of flavonoids in complex samples of grape extracts and wine [28,29]. MRM is a sensitive and highly specific detection method based on the analysis of a highly characteristic fragment ion of the targeted parent ion from the compounds of interest. Skin and seed compositions from three different varieties (Merlot, Syrah and Tannat) were investigated separately.

## 2. Results and Discussion

### 2.1. Identification of Flavanol Glycosides in Grape Skins and Seeds

Flavanol monoglycosides and diglycosides in polyphenol extracts of grape skins and seeds were analyzed, targeting the specific parent ion of each sample and their most characteristic daughter ion fragments. Identification was carried out by comparing retention times and both qualifier and quantifier ions. Ten flavanol monomer glycosides, 10 flavanol monomer diglycosides, 19 flavanol dimer monoglycosides, and three flavanol dimer diglycosides were detected. This is the first time these two diglycoside families in grapes have been reported in grape berries. These compounds may correspond to isomers, with sugar moieties on A, B or C rings, and R or S configuration on the asymmetric carbons producing, once ionized, very specific MS fragmentation patterns as previously described [25,27].

#### 2.1.1. Flavanol Glycoside Monomers

The first objective of this study was to identify the flavanol glycosides isomers present in samples in Merlot, Syrah and Tannat grape skins (sk) or seeds (se), as listed in Table 1. Two types of monomers were screened: flavanol monomers comprising one (glycosides) or two hexose (diglycosides) in their structures.

**Figure 1:**
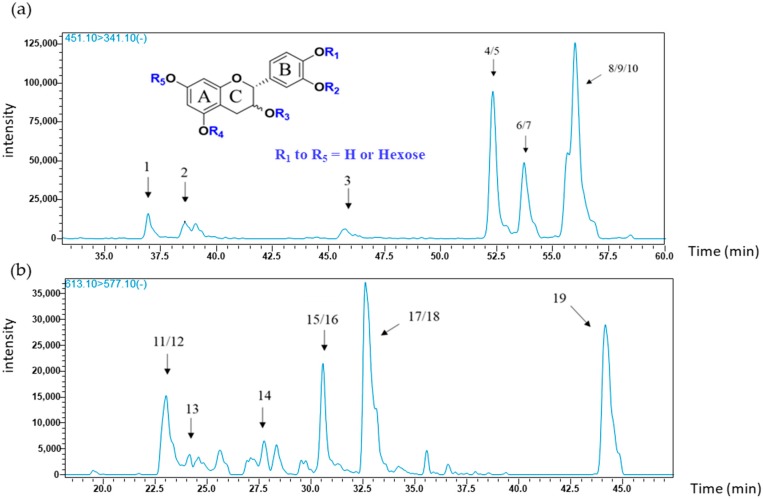
MRM profiles from the UHPLC-MS analysis of (**a**) (epi) catechin glycosides {451 → 341} and (**b**) (epi) catechin diglycosides {613 → 577} of a grape seed extract (Tannat, 25 July).

The principal MS/MS transition observed for flavanol monomer glycosides (*m*/*z* 451), corresponded to the fragment ion at *m*/*z* 341, which results from the loss of the B ring (−110). This was then chosen as the quantifier transition. To further target specific compounds, transitions from qualifier ions were used and are listed in Table 2, (MS/MS product ions); MS^2^ fragment at *m*/*z* 177 correspond to loss of hexose and water, and the *m*/*z* 289 fragment corresponds to the loss of a glucose moiety. Given the location of glucose at one of five possible positions on A, B or C rings, in either the R or S configuration of carbon 3 on ring C, ten possibilities of mono glycosylated (epi) catechin species may be identified. The associated chromatogram extracted from Tannat seed extract of the targeted transition {451 → 341} is presented in Figure 1a. The 10 peaks correspond to all potential isomers of monomeric (epi) catechin glycosides and are presented in Table 2.

Using electrospray ionization quadrupole time-of flight mass spectrometry, Delcambre et al. (2012) identified eight different monomers in Merlot seeds and wine, but none were found in Merlot skins [25]. More recently, five isomers were detected in seeds of three different grape varieties (Syrah, Merlot and Cabernet-Sauvignon), and in several wines made from Tannat, Alicante, Merlot, Syrah and Grenache grapes. Between zero and two isomers were identified in skins depending on grape variety [27]. Based on retention time and characteristic transitions, the C4′OG standard was identified as one of the co-eluting peaks 8, 9 or 10. It may also be observed that all glycosylated flavanol monomers (peaks 1 to 10) were detected in skins and seeds of the three grape varieties at all stages of development, with the exception of peak 2 at 38.6 min. Previous studies showed that grape skins contain a small amount of hexoside monomers during grape ripening, whereas these compounds were not found at maturity [25,27].

In this study, monomeric (epi) catechin diglycosides in grapes are reported for the first time, although flavonoid diglycosides were already described in the literature. Flavanol diglycosides in particular were detected in plums [30,31], and anthocyanin diglycosides, such as cyanidin-3,5-*O*-diglucoside, and malvidine-3,5-*O*-diglucoside in red grape skins and red wines were also reported [32,33]. The chosen quantifier transition corresponds to (epi) catechin diglycosides fragmentation (*m*/*z* 613), producing the fragment at *m*/*z* 577 (double dehydration). The chosen qualifier fragment corresponds to the loss of the two sugar moieties producing a catechin ion (*m*/*z* 289). The superposition of MS/MS chromatograms from each grape variety (seeds and skins) showed nine common peaks with the retention times given in Table 1. An example of the associated chromatograms from the targeted transition {613 → 577} in Tannat seed extract is shown in Figure 1b. The monomeric flavanol diglycosides corresponding to peaks 11 to 19 are all present in all grape seeds.

#### 2.1.2. Flavanol Glycoside Dimers

Two types of (epi) catechin dimers, (epi) catechin dimer monoglycosides and diglycosides were investigated in this study. The following paragraphs explain the common compounds detected in seed and skin grape samples in terms of MS/MS fragments and their related retention times.

**Figure 2:**
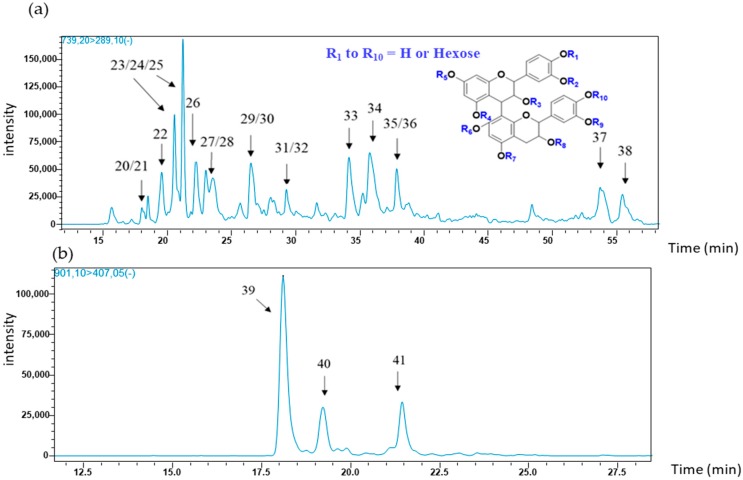
MRM profiles from the UHPLC-MS analysis of (**a**) (epi) catechin dimer glycosides {739 → 289} and (**b**) (epi) catechin dimer diglycosides {901 → 407} of a grape seed extract (Tannat, 25 July).

Dimeric flavanol glycosides (*m*/*z* 739) were investigated for the second time in grapeberries [27]. The most intense transition used for quantification was the fragment at *m*/*z* 739 which yielded an ion fragment at *m*/*z* 289, corresponding to the well-known quinone methide cleavage (QM). Quantifier ions of masses of 177, 287, 339, 451, and 577, were also described in our previous study cited above [27], and as shown in Figure 2a, the chromatogram representing the targeted transition {739 → 289}, we report here 19 flavanol dimer glycosides; their associated retention times and specific fragments are summarized in Table 2. MS^2^ fragments at *m*/*z* 289 would be obtained from a QM without the fragment at *m*/*z* 449, indicating that the hexose position could be attached on the upper unit. MS^3^ fragment at 339 may arise from the loss of the B ring (−110) from the fragment at 449. MS^2^ fragments at *m*/*z* 287 and 451 arise from two potential fragmentations: either a QM indicating that the hexose is possibly positioned on the lower unit, or of heterocyclic ring fission (HRF) with the sugar attached on the upper unit of ring A. The former hypothesis could possibly be indicated by co-elution of two different dimer structures. Previous work showed the presence of both the above compounds in grape seeds and skins, and it was observed that depending on the variety, three to four isomers were detected in seeds, and three isomers in skins [27]. In this study, seed polyphenol extracts from the three varieties contain more flavanol glycoside dimers corresponding to peaks 20 to 38 in Table 2. In skin extracts, they were also observed except peaks 22, 29, 30, 31, 32, 37 and 38 in all grape varieties.

Dimeric (epi) catechin diglycosides (*m*/*z* 901) were investigated for the first time in *Vitis vinifera* grapes. In the literature, a procyanidin dimer-*O*-diglucoside was recently detected in cowpea [23]. Their fragmentation is characterized by an ion with *m*/*z* at 407 (quantifier and qualifier). This fragmentation pattern results from the loss of the two sugar moieties (−162 × 2) followed by a classical Retro Diels Alder fission (−152) and dehydration (−18) [34]. The MRM profile obtained for these compounds from the targeted transition {901 → 407} in Tannat grape seeds is presented in Figure 2b. These three isomers were found in each polyphenol seed extract from the three grape varieties, and were not detected in skin samples in any of the grape varieties.

### 2.2. Quantification of Flavanol Glycosides in Syrah, Tannat and Merlot Grape Skins and Seeds during Ripening

Monomers and dimers of glycosylated flavanols were quantified separately in skins and seeds of Syrah, Tannat and Merlot grapes at different stages of grape development. The ripening of Syrah and Tannat is similar, and consequently in 2017 both were sampled on 28 June, 11 and 25 July, and 14 September. Merlot, on the other hand, ripens earlier and therefore an additional sampling took place on 12 June. Concentrations were expressed in ng per skin or seeds for one berry. The aim was to follow grape ripening, regardless of water or sugar accumulation. The mono- and di-glycosides were separated into two groups (monomers or dimers) and were quantified and semi quantified by MRM using a calibration curve of C4′OG. Changes in skins and seeds were studied separately, and results are discussed in the following two sections.

#### 2.2.1. Changes in Monomeric and Oligomeric Flavanol Glycosides in Grape Skins during Ripening

**Figure 3:**
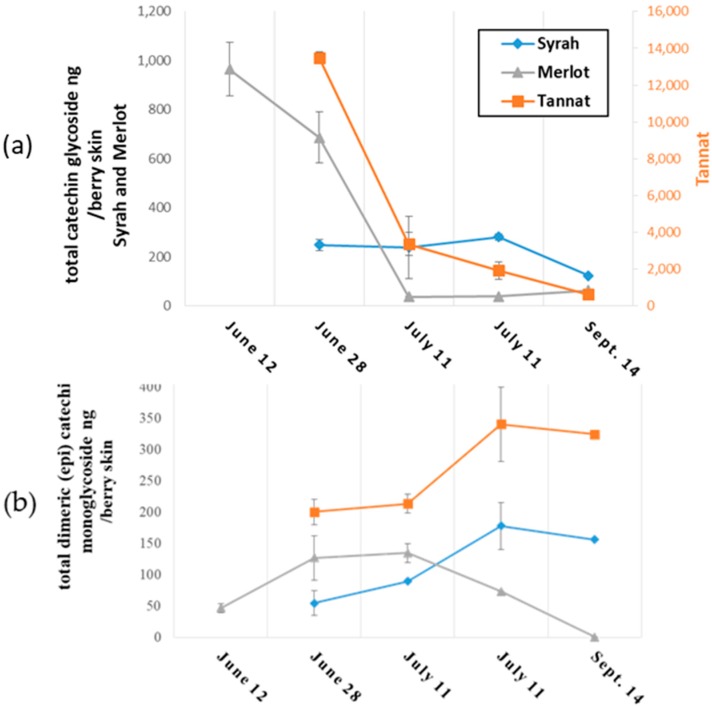
Evolution of (**a**) monomeric (epi) catechin glycosides and (**b**) (epi) catechin dimeric glycosides in grape skins during ripening.

Monomeric and dimeric flavanol glycosides in grape skins from the three varieties were semi quantified, and results are presented in Figure 3a,b, respectively.

Monoglycosylated (epi) catechin monomers are present in all three grape varieties as may be observed in Figure 3a. Greater amounts of the target compounds in polyphenol extracts of skins were found at the start of ripening: their concentrations in Tannat, Merlot and Syrah were approximately 14 mg, 1 mg and 250 ng, respectively. Tannat skins were 10-fold richer in monoglycosides than Merlot skins, and 50 times greater than in Syrah skins. A decrease in the concentration of the compounds was observed during berry development for all skins. Nevertheless, a small increase in monomeric (epi) catechin glycosides was observed in Merlot skins from 25 July to 14 September. These compounds in mature grape skins are reported here for the first time, which may be attributed to the greater detection sensitivity of our method. The concentrations of the glycosides described here are 10^3^ to 10^4^ times lower than the corresponding non-glycosylated flavanol monomers reported in the literature [35].

Changes in the concentrations of (epi) catechin dimer monoglycosides during ripening are presented in Figure 3b. The first observation was that monoglycoside composition evolved in a similar manner for both Tannat and Syrah skins. Up to 25 July, amounts increased to 180 ng per berry skin in Syrah and in Tannat, followed by a slight decrease of about 30 ng between the above date and 14 September. In Merlot skin, the concentration increased to 130 ng per berry skin until 28 June, then stabilized, decreased sharply and were no longer detected after 14 September. The amounts are on average, 100 times lower than values reported for the more widely-known non-glycosylated procyanidines (B1, B2, B3 and B4) in grape skins [36].

To summarize, Merlot, Syrah and Tannat skins were found to contain mono-glycosylated flavanol monomers and dimers; at the earlier stages of development, monomers occurred in greater concentrations than dimers in skins, whereas at maturity, concentrations of both monomer and dimer and glycosides were similar. Overall, total concentrations of (epi) catechin glycosides were significantly higher in Tannat than in Syrah and Merlot grape skins at all sampling dates.

#### 2.2.2. Evolution of Monomeric and Oligomeric Flavanol Glycosides in Grape Seeds during Ripening

**Figure 4:**
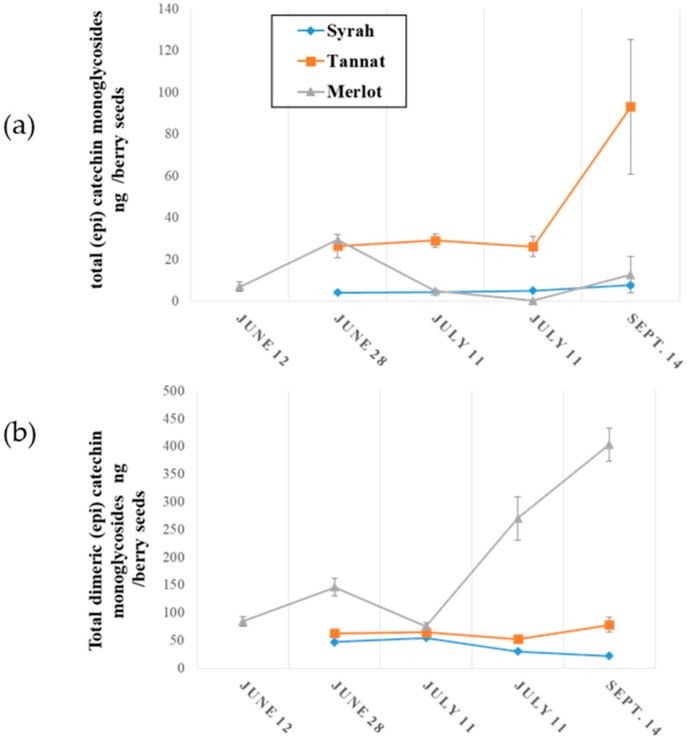
Evolution of (**a**) monomeric (epi) catechin monoglycosides and (**b**) dimeric (epi) catechin monoglycosides during grape seed ripening.

The evolution of monomeric and oligomeric flavanol glycosides in grape seeds during ripening is illustrated in Figure 4.

The concentrations of monomeric (epi) catechin glycosides in grape seed samples during ripening are presented in Figure 4a where it might be observed that concentrations were higher in Tannat grape seeds (up to 80 ng per berry seeds) and their concentrations increased during development. A small variation was observed for Merlot with an increase to 30 ng per berry in 28 June, followed by a decrease to 12 ng per berry between the above date and 14 September. The quantity of (epi) catechin glycosides in Syrah seeds remained constant during ripening at approximately 7 ng per berry. These concentrations are 10^3^ to 10^4^ times lower than those of non-glycosylated monomeric flavanols as reported in the literature [32,36].

Higher concentrations of (epi) catechin dimer glycosides in grape seeds were generally observed in all varieties as seen in Figure 4b. As above, amounts increased during grape development, and were, at the end of ripening (14 September), 400 ng, 80 ng and 20 ng per berry in Merlot, Tannat and Syrah seeds, respectively. These amounts are approximately 10-to-100 times greater than those of procyanidines (B1, B2, B3 and B4 types) in grape seeds [36].

To summarize, both (epi) catechin monomer and dimer flavanol monoglycosides present in seeds samples would appear to increase during berry development. In addition, mature grape skins would appear to contain more (epi) catechin monomer glycosides than in mature seeds.

**Figure 5:**
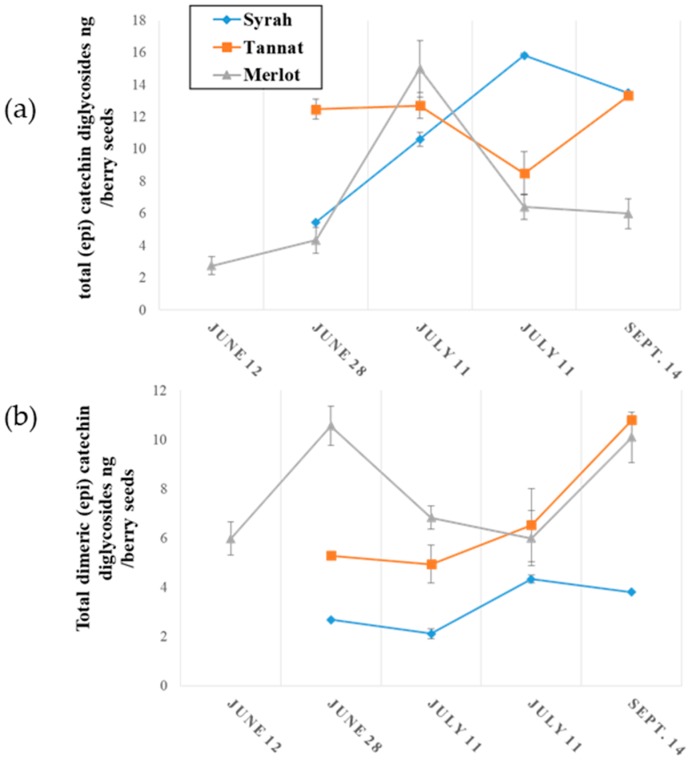
Evolution of (**a**) (epi) catechin diglycosides and (**b**) (epi) catechin dimer diglycosides during grape seeds ripening.

Finally, for the first time the evolution of (epi) catechin monomer and dimer diglycosides in grapes are described. As already stated, these compounds were only detected in grape seeds and their variation during ripening is illustrated in Figure 5.

Figure 5a represents the change in diglycosylated (epi) catechin monomers during ripening An accumulation was observed for Merlot and Syrah seeds, with a greater increase for Syrah. From 28 June to 14 September, monomer diglycosides increased by 3 ng in the seeds of one berry for Merlot, and 6 ng per berry seeds for Syrah. From the onset to the end of ripening, the concentrations of (epi) catechin diglycosides remained stable at around 12 ng per berry seeds.

As with monomers, the amount in (epi) catechin dimer diglycosides increased during seed development for the three grape varieties represented in Figure 5b. Their concentrations increased by about 4 ng per berry seeds for Merlot and Tannat, and 2 ng per berry seeds for Syrah from the start to the end of the development. Diglycoside quantities were similar among all three varieties.

To summarize, monomeric and dimeric flavanol mono- and diglycosides were found only in grape seeds, and higher concentrations were observed for monoglycosides than for diglycosides in the three varieties studied.

## 3. Materials and Methods

### 3.1. Reagents, Standards and Calibration

Deionized water was purified with a Milli-Q purification system (Millipore, Molsheim, France). Acetone and diethyl ether were obtained from Analytic Lab (St. Mathieu de Treviers, France). Formic acid and HPLC grade methanol were purchased from Sigma Aldrich (St. Louis, MO, USA). (+)-Catechin was purchased from Sigma Chemical Company (St. Louis, MO, USA). (+)-Catechin 4′-*O*-β glucoside (C4′OG) was hemi-synthesized as described previously [27].

A calibration curve of C4′OG was prepared in the ranges 0.025 to 5 mg·L^−1^ in a mixture of methanol/water (5:5). Flavanol diglycosides (monomers, dimers) were semi-quantified by MRM with, C4′OG as standard. The limits of detection and quantification, based on the C4′OG calibration curve were respectively of 0.03 ng/L and 0.11 ng/L.

### 3.2. Grapes

#### 3.2.1. Samples used for Quantification of Flavanol Glycosides in Berry Skin and Seeds

**Figure 6:**
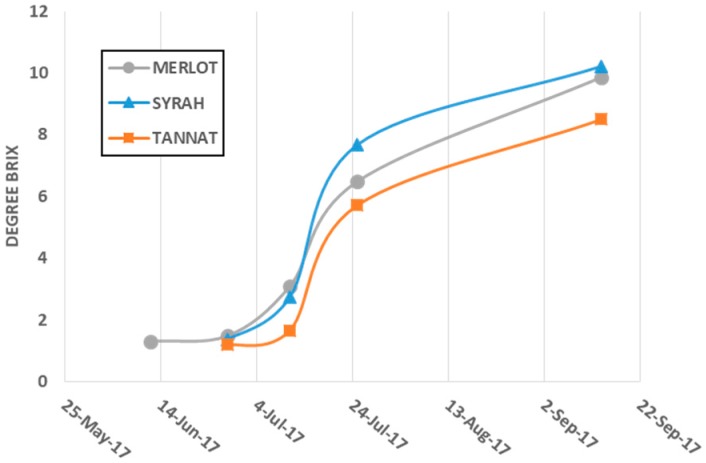
Degree brix evolution of Merlot, Syrah and Tannat grape during ripening.

The grapes of the three *V. vinifera* varieties from the same vineyard were harvested at four different dates for Syrah and Tannat, and five dates for Merlot, corresponding to different developmental stages in May, July and September 2017. For each variety, 3 × 30 fresh berries were randomly selected and weighed. Seeds and skins were removed by hand on ice and weighed separately. Another set of 100 berries was crushed and its average sugar concentration was determined using a hand-held refractometer, and expressed as brix degrees, the evolution of which is shown in Figure 6. Evolution of berry, seed and skin weights during development are summarized in Appendix A.

#### 3.2.2. Extraction of Grape Polyphenols

Grape skins and seeds were extracted separately overnight using 200 mL of acetone/water 7:3 in 1 L bottles under nitrogen with mechanical mixing. Solutions were filtered on cellulose filter paper and evaporated with a rotary evaporator to dryness under reduced pressure at 37 °C. The residues were dissolved in deionized water, frozen at −80 °C and lyophilized with a Cryotec^®^ lyophilizer, and the resulting tannin extract powders were stored at −20 °C, until further analysis.

### 3.3. Optimization of a UHPLC-ESI-QQQ-MS/MS in Multiple Reaction Monitoring (MRM) Method for Quantification of Flavanol Glycosides

#### 3.3.1. UHPLC Analysis

The chromatographic apparatus consisted of Nexera X2 UHPLC system (Shimadzu, Marne la Vallée, France) equipped with a binary pump, solvent degasser, and thermostatted column compartment. Two reversed-phase columns in series were used for separation: Zorbax SB AQ (2.1 × 150 mm and 2.1 × 100 mm 1.8 μm from Agilent Technologies, Santa Clara, CA, USA). Mobile phase A and B consisted of water (0.1% formic acid) and methanol (0.1% formic acid) respectively. The 77 min linear gradient program used was 0% B for 7 min, 0–18% B over 10 min, 18–33% B over the next 40 min, followed by 40–100% B for 1 min. A plateau at 100% B for 9 min was used to wash the column, decreasing from 100–0% B in 1 min, followed by a 9-min post-run isocratic step at 0% B to re-equilibrate the column. The flow rate was constant at 0.35 mL/min at 40 °C.

#### 3.3.2. MS/MS Detection

MS/MS experiments were carried out using the Shimadzu UHPLC system described above coupled to a Shimadzu LCMS-8050 triple quadrupole mass spectrometer using the multiple reaction monitoring (MRM) technique operating in negative ion mode. The following parameters were used for all experiments: electrospray interface voltage was 3 kV; heat block temperature of 350 °C; desolvation line temperature of 300 °C; interface temperature of 300 °C; drying gas flow gas was set at 5 L/min; and nebulizing gas flow at 3 L/min, heating gas flow rates were set at 15 L/min. Q1 and Q3 were set to unit resolution. The dwell time was set at 1 ms for each MRM transition; optimal CE values were chosen for each class of compounds to obtain the most characteristic fragments (Table 2). The followed compounds were targeted: (epi) catechin monomer glycosides, **1**; (epi) catechin dimer glycosides, **2**; (epi) catechin monomer diglycosides, **3**; (epi) catechin dimer diglycosides, **4**.

## 4. Conclusions

Monomeric and dimeric flavanol monoglycosides and diglycosides were shown to be present in grapes from three different *Vitis vinifera* varieties (Merlot, Syrah and Tannat). An original UHPLC-MRM method allowed the study of changes in the concentrations of these compounds during grape ripening. Grape seed extracts contained monomeric and dimeric flavanol mono- and diglycosides. With the exception of diglycosylated flavanol dimers, these compounds were also detected in grape skin extracts. The concentration of the mono and diglycosides depended largely on both grape variety and whether they occurred in grape skins or seeds. The amounts of monoglycosylated flavanols varied from 10 to 100 ng per seeds for one berry, and from 60 to 600 ng per skin for one berry at maturity. Monoglycosylated flavanol dimers quantities ranged from 20 to 400 ng per seed and 0 to 300 ng per berry skin. Seeds contained on average from five to 15 ng of monomeric flavanol diglycosides and from three to 10 ng dimeric flavanol diglycosides. The amounts of monoglycosylated flavanols in grape seeds were greater than those of diglycosylated flavanols.

Flavanol monomer monoglycosides decreased progressively during grape ripening for all grape and skin extracts. Conversely, flavanol glycoside concentrations seemed to increase before *veraison* and thereafter to decrease and depended on the grape variety. In light of the results presented in this study, the precise structures and the role of these new compounds in proanthocyanidin biosynthesis in grapes or in other plants warrant further investigation.

## Figures and Tables

**Table 1:** (Epi) catechin glycosides isomer peaks with their related retention time and fragmentation pattern from MS/MS analysis.

Peaks	RT (min)	Tissues, Seeds (se), Skins (sk)	MS/MS Product Ions *m*/*z*	Compounds (Corresponding *m*/*z*)
1	36.95	se, sk	177; 289; 341	(epi) catechin monoglycoside (451)
2	38.60	sk	177; 341	
3	45.72	se, sk	177; 341	
4/5	52.33	se, sk	177; 289; 341	
6/7	53.77	se, sk	177; 289; 341	
8/9/10	55.99	se, sk	177; 341	
11/12	23.33	se	577; 289	(epi) catechin diglycoside (613)
13	24.15	se		
14	27.74	se		
15/16	30.57	se		
17/18	32.64	se		
19	44.17	se		
20/21	18.48	se, sk	177; 287; 289; 451; 577	(epi) catechin dimer monoglycoside (739)
22	19.54	se	177; 287; 289; 451; 577	
23/24/25	21.20	se, sk	177; 287; 289; 339; 451; 577	
26	22.21	se, sk	177; 287; 289; 339; 451; 577	
27/28	23.52	se, sk	177; 287; 289; 339; 451; 577	
29/30	26.17	se	177; 287; 289; 339; 451; 577	
31/32	28.04	se	177; 289; 451; 577	
33	34.13	se, sk	177; 287; 289; 339; 451; 577	
34	35.75	se, sk	177; 287; 289; 339; 451; 577	
35/36	37.85	se, sk	177; 289; 339; 451; 577	
37	48.39	se	287; 289; 339; 451	
38	53.73	se	287; 289; 339; 451; 577	
39	18.09	se	407	(epi) catechin dimer diglycoside (901)
40	19.25	se		
41	21.43	se		

**Table 2:** MRM parameters for each (epi) catechin glycosides derivatives.

Compound	Precursor Ion	Product Ion	Q1 Prebias	Collision Energy (eV)	Q3 Prebias
**1**	451	341	22	18	25
**2**	739	289	40	31	26
**3**	613	577	30	12	22
**4**	901	407	24	49	14

**Figure A1:**
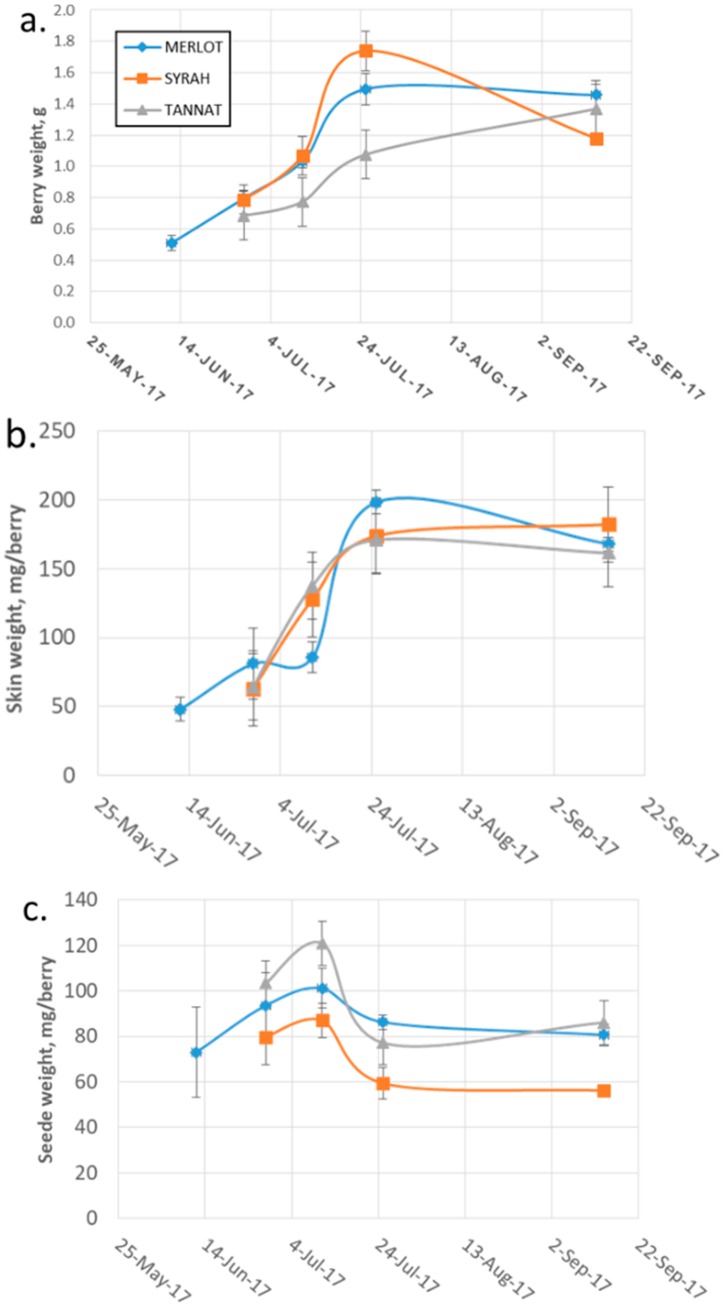
Evolutions of (**a**) berry weight (g), (**b**) skin weight (mg/berry) and (**c**) seed weight (mg/berry) during berry development.
